# Abstracts

**DOI:** 10.1155/S1463924682000157

**Published:** 1982


Journal of Automatic Chemistry, Volume 4, Number 2 (April–June 1982), pages 51–53

### Abstracts

**Page 54**
**Computer software for a simultaneous multi-element atomic-absorption spectrometer**
J. M. Harnly *et al.*

A unique multi-element atomic-absorption spectrometer, with a continuum source (SIMAAC), has been developed using a PDP 11/34 Digital Equipment Corporation minicomputer. In addition to a multi-element capability SIMAAC has unique analytical features. This is brought about to some degree by the computing facilities. The computer is used for data acquisition, analytical calculations, prompting the analyst in the operating procedure, and long-term data file storage. The programs are highly versatile, incorporating a statistical approach in the analytical calculations. The detailed operation of three computer modules is described and the execution time for a run of samples is outlined. A copy of the software routines is available from the authors on request. The complete system is compatible with automatic sample injection, either using flame or electrothermal atomization.

**Un spectromètre pour absorption atomique automatisé pour l'analyse ultiélément**

J. M. Harnly *et al.*

Un nouvaux spectromètre à absorption atomique multiélément avec une source continue (SIMAAC) qui utilise un PD11/34 de la Digital Equipment Corporation est décrit. En plus de la capacité de l'analyse multiélément, le SIMAAC contient de nouvelles propriétés analytiques. Celles-ci sont en partie réalisables grâce à la capacité de calcul du miniordinateur. La calculatrice est utilisée pour la réception des données, le calcul analytique, elle guide également l'analyste pendant la manipulation de l'instrument et est utilisée pour archiver les données. Les programmes sont très flexibles et utilisent un procédé statistique pour le calcule des résultats de l'analyse. Le fonctionement des trois modules de calculateur est décrit d'une façon détaillée et le temps d'analyse pour une série d'échantillons est décrit. Une copie du programme sera envoyée par l'auteur sur demande. Le système complet est équipé d'une injection d'échantillon automatisée et est compatible avec des systèmes à atomisation par flammes ou électrothermique.

**Ein automatisiertes Atomabsorption-Spektrometer für die Multielement-Analyse**
J. M. Harnly *et al.*

Ein neuartiges Multielement Atomabsorption-Spektrometer mit einer kontinuierlichen Quelle (SIMAAC), das einen PDP11/34 Digital Equipment Corporation Minirechner benützt, ist beschrieben. Zusätzlich zur Fähigkeit der Multielement-Analyse weist SIMAAC neuartige analytische Eigenschaften auf. Diese werden zum Teil durch die Rechenleistung ermöglicht. Der Rechner wird für die Datenerfassung, die analytischen Berechnungen, die Führung des Analytikers in der Durchführung und Bedienung und für die Archivierung der Daten benützt. Die Programme sind sehr flexibel und verwenden ein statistisches Vorgehen für die Berechnung der Analysenresultate. Die detailierte Funktionsweise der drei Rechnermodule ist angeführt und die Durchführungszeit für eine Serie von Proben ist beschrieben. Eine Kopie der Programme wird vom Autor auf Wunsch abgegeben. Das gesamte System ist mit automatisierter Probeneinspritzung auf der Basis von Flammen- oder elektrothermischer Atomisierung kompatibel.

---

**Page 61**
**An automated module for atomic-absorption analysis of reduced volatile elements**
B. W. Renoe

The design of a module for partial automation of the sodium borohydride reduction procedure for determining easily volatilized elements (such as arsenic and mercury) by atomic-absorption spectrometry is described. The device incorporates valves to control the direction of purge gas flow, the addition of reagent acid and the addition of borohydride reductant. Timing and logic circuitry provide a precise sequence of analytical events (purge gas flow, triggering of the data acquisition, sample acidification, metal reduction, and final system reset). This module, coupled to an atomic-absorption instrument, can provide improved precision, and a streamlined analytical protocol for the routine determination of easily volatilized elements.

**Module automatique pour analyse par absorption atomique d'éléments volatiles réduits**

B. W. Renoe

La construction d'un module est décrite, qui permet l'automation partielle de la méthode de reduction par sodium borohydride pour la détermination automatique d'éléments volatiles tels que l'arsène et le mercure par spectrométrie par absorption atomique. L'instrument comprend le contrôle des vannes pour le débit de gaz, l'ajout d'acide et du réducteur borohydride. Les circuits logiques et les temps d'attente permettent une sequence précise des pas analytiques (débit de gaz de blayage, activation du transfer de données, acidification de l'échantillon, réduction métallique et retour à l'état initial pour le prochain échantillon). Ce module permet en combinaison avec un instrument pour l'absorption atomique une meilleure précision et un compte rendu clair pour l'analyse de routine d'élements facilement volatilisés.

**Ein automatisches Modul für Atomabsorbtions-Analysen reduzierter, flüchtiger Elemente**

B. W. Renoe

Es wird die Konstruktion eines Moduls beschrieben, das der teilweisen Automation des Natriumborhydrid-Reduktionsverfahrens zur Bestimmung leicht flüchtiger Elemente wie Arsen und Quecksilber durch Atomabsorbtions-Spektrometrie dient. Das Gerät umfasst die Steuerung der Ventile für den Spülgasfluss, die Zugabe von Säure und von Borhydrid-Reduktionsmittel. Zeit- und Logikschaltung erlauben eine präzise Sequenz der analytischen Schritte (Spülgasfluss, Auslösen der Datenübernahme, Ansäurung der Probe, Metallreduktion, und Erstellung des Ausgangszustandes für die nächste Probe). Dieses Modul ermöglicht in Verbindung mit einem Atomabsorbtions-Instrument verbesserte Präzision und ein rationelles Analysenprotokoll für die Routinebestimmung leicht flüchtiger Elemente.

---

**Page 65**
**Automated determination of 5-hydroxyindolylacetic acid in urine by high performance liquid chromatography**
J. P. Garnier *et al.*

In carcinoid tumour detection, the measurement of 5-hydroxyindolylacetic acid (5-HIAA) is an indispensable adjunct to blood 5-hydroxytryptamine determination. Such measurement must be systematic and needs a fast and reliable technique. For this purpose, high-performance liquid anion exchange chromatography is combined with continuous flow spectrofluorometry at an optimized pH. The authors use an automated injector, thus the technique is completely automatic. Its chief advantages are rapidity (7 min.

per determination), enabling measurement of large series of samples, precision (CV = 0·72%), sensitivity (detection limit: 1 pmol) and specificity (there is no interference with 5-HIAA precursors or with the drugs tested by the authors). Reference values ($N = 112$) are 5 ± 20 μmol/24 h. Comparison with a reference spectrofluorometric technique shows a correlation coefficient of 0·96. When the present method, which requires no prior extraction, was compared to a specific ethyl acetate extraction method, the correlation coefficient was 0·99.

**Détermination automatisée de 5-hydroxy-indolyl-acide acétique dans l'urine à l'aide de la chromatographie liquide à haute performance**

J. P. Garnier *et al.*

Dans l'examen d'une tumeur cancéreuse, la mesure de 5-hydroxyindolyl acide acétique (5-HIAA) doit s'adjoindre à la détermination de 5-hydroxytryptamine dans le sang. Ces mesures doivent être pratiquées systématiquement et demandent une technique sûre et rapide. Dans ce but, la chromatographie liquid à haute performance par échange d'anions est combinée avec la spectrofluorométrie à flux continu à une valeur optimale de pH. Les auteurs utilisent une injection automatique. Avec cela, la technique est complètement automatisée. Les avantages principaux sont la vitesse (7 min. par détermination) rendant possible la mesure d'une grande série d'essais répétitifs, la réproductibilité (CV = 0·72%), la sensibilité (limite de la détection: 1 pmol) et la spécifité (pas d'interférence avec des stades antérieurs de 5-HIAA ou avec les drogues examinées par les auteurs). Les valeurs de référence ($N = 112$) sont 25 ± 20 μmol/24 heures. La comparaison avec une méthode spectrofluorométrique de référence présente un coefficient de corrélation de 0·96. Quand la méthode proposée, qui demande aucune extraction précédente, est comparée à une méthode spécifique d'extraction d'acétate éthylique, le coefficient de corrélation égale à 0·99.

**Automatisierte Bestimmung von 5-Hydroxyindolyl Essigsäure in Urin mit Hilfe HP-Flüssigchromatographie**
J. P. Garnier *et al.*

Bei der karzioniden Tumorprüfung ist die Messung von 5-Hydroxyindolyl Essigsäure (5-HIAA) ein unerlässlicher Zusatz zur Blut 5-Hydroxytriptamin Bestimmung. Solche Messungen müssen systematisch durchgeführt werden können und verlangen eine schnelle, zuverlässige Technik. Zu diesem Zweck wird die Hochleistungs-Anionenaustausch-Flüssigchromatographie kombiniert mit der Spektrofluorometrie bei optimalem pH im kontinuierlichem Fluss. Die Autoren benützen eine automatisierte Einspritzung—die Technik ist somit völlig automatisch. Die Hauptvorteile sind Schnelligkeit (7 Min. pro Bestimmung) ermöglichen der Messung von grossen Probenserien, Wiederholbarkeit (CV = 0·72%), Empfindlichkeit (Empfindlichkeitsgrenze: 1 pmol) und Spezifität (keine Störung durch 5-HIAA Vorläufer oder mit Drogen, die durch die Autoren ausgeprüft wurden). Referenzwerte ($N = 112$) sind 25 ± 20 μmol/24 h. Der Vergleich mit einer specktrofluorometrischen Referenzmethode zeigt einen Korrelationskoeffizienten von 0·96. Wird die vorliegende Methode, welche keine vorgängige Extraktion effordert, mit einer spezifischen Aethylacetat-Extraktionsmethode vergliche, beträgt der Korrelationskowffizient 0·99.

---

**Page 69**
**The use of Fuller's earth to reduce acetoacetate interference in the measurement of serum creatinine by the Beckman Astra-4 analyser**
J. Thompson

Acetoacetate produces a positive interference in the measurement of serum creatinine by the Beckman Astra-4 analyser. Sera enriched with acetoacetate to give acetoacetate concentrations of 4 mmol/l and 10 mmol/l showed apparent mean increases of 131 and 347 μmol/l in measured creatinine. The procedure described involves the use of Fuller's earth to absorb creatinine in serum. The absorbed creatinine is eluted from the Fuller's earth under alkaline conditions, and measured on the Astra-4. The procedure reduces the interference by acetoacetate at serum concentrations of 4 mmol/l and 10 mmol/l to apparent mean increases of 2 and 12 μmol/l in measured creatinine. The procedure is quick and simple to perform and requires 200 μl of serum.

**L'utilisation de terre-Fuller pour la réduction de l'interférence acéto-acétate lors du dosage de créatinin de sérum avec l'analysateur Astra-4 de Beckman**

J. Thompson

Acétoacétate produit une déviation positive de la mesure de la créatinin de sérum dans l'analyseur Astra-4 de Beckman. Des sérums enrichis avec acétoacétate avec des concentrations de 4 mmol/l et 10 mmol/l montrent des moyens apparemment enlevés de 131 et 347 μmol/l en créatinin mesuré. Le procédé décrit utilise la terre-Fuller pour l'absorption du créatinin dans le sérum. Le créatinin absorbé est lavé sous conditions alcalines et mesuré dans l'Astra-4. Cette procédure réduit l'influence de l'acétoacétate lors de concentrations de sérum de 4 mmol/l et 10 mmol/l à une élévation du moyens apparemment en créatinin mesuré de 2 et 12 μmol/l. La procédure est rapide et facile a exécuter et nécessite 200 μl de sérum.

**Die Verwendung von Fuller-Erde für die Reduktion der Acetoacetat-Interferenz bei der Bestimmung von Serumkreatinin mit dem Beckman Astra-4 Analysator**
J. Thompson

Acetoacetat verursacht eine positive Beeinflussung der Messung von Serumkreatinin im Beckman Astra-4 Analysator. Mit Acetoacetat angereicherte Seren, die Acetoacetat-Konzentrationen von 4 mmol/l und 10 mmol/l ergeben, zeigten ʼscheinbare Mittelwertüberhöhungen an gemessenem Kreatinin von 131 und 347 μmol/l. Das beschriebene Vorgehen besteht aus der Verwendung von Fuller-Erde zur Adsorption des Kreatinin im Serum. Das adsorbierte Kreatinin wird unter alkalischen Bedingungen aus der Fuller-Erde ausgewaschen und im Astra-4 gemessen. Die Vorschrift reduziert den Einfluss von Acetoacetat bei Serumskonzentrationen von 4 mmol/l und 10 mmol/l auf eine scheinbare Mittelwertsüberhöhung an gemessenem Kreatinin von 2 und 12 μmol/l. Die Vorschrift ist rasch und einfach durchzuführen und erfordert 200 μl Serum.

---

**Page 72**
**Assessment of the validity of error flags generated by the Technicon SMAC system and Perkin-Elmer KA 150 enzyme analyser**
E. A. Robertson *et al.*

The Technicon SMAC and Perkin-Elmer KA 150 enzyme analyser print error flags to indicate certain results are unreliable because of unacceptable conditions detected during the analysis. The validity of these error flags was assessed by repeat analyses of multiple specimens from each of 10 volunteers. For various SMAC tests the percentage of flagged results ranged from 0·4–10 and for the KA 150 from 0·2–11. The deviation of all results for a particular determination from the mean of the subject's unflagged results was calculated. The mean deviations of flagged values was significantly different from that of unflagged values for 14 of 19 SMAC tests and two of five KA 150 tests. The variance of the deviation values was significantly greater for flagged results for 17 SMAC and three KA 150 assays. Large departures from the unflagged mean were significantly more frequent for flagged results for 17 SMAC and all KA 150 tests.

**Etude de la valideté des marques d'erreur générées par le système Technicon SMAC et l'analyseur pour enzymes Perkin-Elmer KA 150**

E. A. Robertson *et al.*

Le SMAC de Technicon, ainsi que l'analyseur d'ensymes Perkin-Elmer KA 150 impriment des marques d'erreur pour indiquer que certains résultats sont douteux à cause de conditions inadmissibles détectées pendant l'analyse. La validité de ces marques d'erreur a été vérifiée par analyses répétées d'échantillons multiples de dix volontaires. Pour différent tests avec le SMAC, le pourcentage des résultats marqués variait de 0·4 à 10% et pour le KA 150 de 0·2 à 11%. La déviation de tous les résultats pour une détermination particulière de la moyenne de tous les résultats non marqués a été calculée. Les déviations moyennes des résultats marqués déviaient d'une façon significative de celles des résultats non marqués, pour 14 des 19 tests SMAC et pour 2 des 5 tests KA 150. La variance des déviations était significativement plus grande pour les résultats marqués pour 17 tests SMAC et 3 tests KA 150. Le nombre des déviations importantes de la moyenne non marquée était significativement plus fréquent pour les résultats marqués de 17 tests SMAC et pour tous les tests KA 150.

**Beurteilung der Gültigkeit von Fehlermarken, die durch das Technicon SMAC System und den Perkin-Elmer KA 150 Enzym-Analysator generiert werden**
E. A. Robertson *et al.*

Das Technicon SMAC System und der Perkin-Elmer KA 150 Enzym-Analysator drucken Fehlermarken aus, um damit anzudeuten, dass gewisse Resultate unzuverlässig sind, weil während der Analyse untolerierbare Bedingungen festgestellt wurden. Die Gültigkeit dieser Fehlermarken wurden durch wiederholte Analysen multipler Proben von 10 verschiedenen Quellen beurteilt. Der Prozentsatz der markierten Resultate varierte für verschiedene SMAC Analysen zwischen 0·4 und 10 und für den KA 150 zwischen 0·2 und 11. Daraufhin wurde die Abweichung aller Resultate einer bestimmten Analyse vom Mittelwert der unmarkierten Resultate berechnet. Die mittlere Abweichung der markierten Werte war signifikant verschieden von den unmarkierten Werten für 14 der 19 SMAC Analysen und für zwei der fünf KA 150 Analysen. Die Varianz der Abweichungswerte war signifikant grösser für markierte Resultate für 17 SMAC und drei KA 150 Analysenmethoden. Grosse Abweichungen vom Mittelwert der unmarkierten Resultate waren signifikant häufiger für die markierten Resultate für 17 SMAC und für alle KA 150 Analysenmethoden.

---

**Page 75**
**A compact automated titration system, applied to a high-precision determination of calcium in sea-water**
L. Anderson and A. Granéli

A titration system built up around a Commodore PET 2001 personal computer is used for photometric titration of calcium in sea-water. The titration unit consists of a modified Metrohm motor burette, acting as measuring device, titration vessel and cuvette at the same time. The photometer used is a Brinkman PC/600 Colorimeter; light is guided between the colorimeter and the titration unit by fibre optics. The output signal of the colorimeter is converted to digital form by a HP 3436A digital multimeter, having an IEEE-488 bus output. The problems when connecting a PET 2001 computer with external devices, using the PET IEEE-488 bus, is described in detail. The calcium titration is performed at pH 8·6 using EGTA as titrant. The decrease of calcium is followed by the simultaneous titration of zinc, using zincon as indicator. Evaluation is carried out by means of a Gran function, using the absorbence and the volume of titrant as data. When the described titration system was used on board a research ship, the calcium titration gave a precision, expressed as coefficient of variation, of 0·16%.

**Utilisation d'un système de titrage automatique et compacte pour le dosage précis du calcium dans l'eau de mer**
L. Anderson and A. Granéli

Un système de titrage conçu autour du miniordinateur Commodore PET 2001 est utilisé pour le titrage photometrique du calcium dans l'eau de mer. Le système de titrage consiste d'une burette à moteur Metrohm et d'un vase de titrage qui sert en même temps de cuvette. Un colorimètre Brinkmann PC/600 sert de photomètre. La lumière est transmise par fibres optiques vers l'unité de titrage. Le signal du colorimètre est digitalisée par un multimètre digital HP 3436A avec une sortie bus IEEE-488. Les problèmes qu'on encontre lors de la connection d'un PET 2001 avec instruments externes à l'aide d'un PET IEEE-488 bus sont décrits en détail. Le titrage du calcium est effectué avec de l'EGTA à un pH de 8.6. La diminutiondu calcium est controlée à l'aide d'un titrage simultané du zinc en utilisant du zincon comme indicateur. L'evaluation est faite d'après la méthode de Gran utilisant comme données l'absorption et le volume de titrant. Le système de titrage a été utilisée à bord d'un bateau de recherche et a permis de déterminer le calcium avec une haute précision et un coefficient de variance de 0.16%.

**Verwendung eines kompakten automatischen Titrationssystem zur hochpräzisen Bestimmung von Kalzium in Meerwasser**
L. Anderson and A. Granéli

Ein um einen Commodore PET 2001 Kleincomputer aufgebautes Titrationssystem wird für die photometrische Titration von Kalzium in Meerwasser benützt. Die Titrationseinheit besteht aus einer modifizierten Metrohm Motorkolbenbürette, die der Messung dient, und einem gleichzeitig als Küvette verwendeten Titrationsgefäss. Als Photometer wurde ein Brinkmann PC/600 Kolorimeter benützt dessen Licht über Glasfaseroptik zur Titrationseinheit geführt wird. Das Ausgangssignal des Kolorimeters wird durch ein HP 3436A Digitalmultimeter digitalisiert und an einem IEEE-488 Bus zur Verfügung gestellt. Die Probleme, die beim Verbinden eines PET 2001 Rechners mit externen Geräten über den PET IEEE-488 Bus entstehen, werden im Detail beschrieben. Die Kalziumtitration wird unter Verwendung von EGTA als Titriermittel bei einem pH von 8.6 durchgeführt. Die Abnahme von Kalzium wird verfolgt durch die gleichzeitige Titration von Zink, wobei Zincon als Indikator verwendet wird. Die Auswertung erfolgt mit Hilfe einer Granfunktion, worin die Absorption und das verbrauchte Volumen des Titriermittels als Daten eingehen. Als das beschriebene Titrationssystem an Bord eines Forschungsschiffes verwendet wurde, ergaben die Kalziumtitrationen eine Präzision, ausgedrückt als Variationskoeffizient, von 0·16%.

